# The political dimensions of rewilding preference

**DOI:** 10.1016/j.isci.2025.113349

**Published:** 2025-09-03

**Authors:** Marek Giergiczny, Rowan Dunn-Capper, Wiktor Budziński, Nestor Fernandez, Henrique M. Pereira

**Affiliations:** 1 German Centre for Integrative Biodiversity Research (iDiv) Halle-Jena-Leipzig, Puschstrasse 4, 04103 Leipzig, Germany; 2 Faculty of Economic Science, University of Warsaw, ul Długa 44/50, 00-241 Warsaw, Poland; 3 Institut für Biologie, Martin-Luther-University Halle-Wittenberg, Halle, Germany; 4 CIBIO (Research Centre in Biodiversity and Genetic Resources)–InBIO (Research Network in Biodiversity and Evolutionary Biology), Universidade do Porto, Vairão, Portugal

**Keywords:** Environmental science, Nature conservation, Social sciences, Political science

## Abstract

Rewilding is increasingly recognized in Europe as a strategy for ecological restoration. However, there has been limited study of the socio-political dimensions of rewilding projects, particularly how political affiliation may influence preferences for rewilding. Given the turbulent European political landscape and significant new environmental legislation, such as the European Green Deal and Nature Restoration Regulation, understanding the interaction between politics and rewilding preferences is critical. We employed a discrete choice experiment approach to assess the impact of political ideology on respondents’ willingness to pay for rewilding interventions in the Oder Delta, which spans evenly across Germany and Poland. Our findings indicate that politics is a major driver of rewilding preferences in both countries, and while rewilding is favored across party lines, the extent of preference is often aligned with the left-right political spectrum.

## Highlights

• Political affiliation predicts support for rewilding interventions
• Left-leaning voters generally show higher willingness-to-pay for rewilding
• Support for rewilding varies by landscape type and party ideology
• Voting preference explains more variation in willingness-to-pay than socio-demographics

## Graphical abstract

**Figure:**
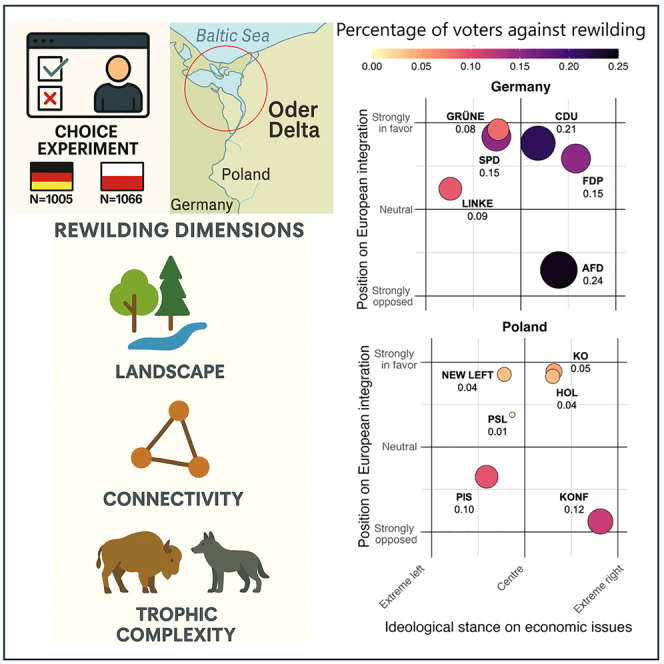

## Introduction

To address the interlinked threats of climate change and biodiversity loss, governments worldwide are implementing extensive conservation programs to conserve and restore habitats. However, the success of these initiatives is frequently entangled with politics; environmentalism has become a contentious political issue, and a central element in the left-right division.^1^ With political polarization potentially hindering the implementation of environmental policy,^2^ it is critical to understand the factors that influence real-world conservation behaviors. This understanding is vital to develop conservation policies and management strategies that are both effective and politically viable.^3^

Previous research has established a significant relationship between political affiliation and environmental values.^4^^,^^5^^,^^6^ Environmentalism often aligns along the liberal-conservative political spectrum.^1^ Typically, left-leaning voters exhibit greater environmental concern,^5^ prioritizing nature conservation over traditional land uses, such as hunting and grazing livestock.^4^^,^^7^ In contrast, conservative groups are increasingly resistant to environmental protection measures,^8^ a trend evident in climate change discourse,^9^^,^^10^ and wider environmental policy.^11^

Rewilding is gaining prominence in Europe as a strategy for ecosystem restoration, attracting attention in popular discourse and at the policy level.^12^^,^^13^ Unlike conventional restoration methods that manage ecosystems on a trajectory toward a specific desired end state,^14^^,^^15^ rewilding seeks to establish self-sustaining ecosystems, which often yield uncertain and dynamic outcomes.^16^^,^^17^^,^^18^ Existing on a spectrum of scale, connectivity, and level of human input,^18^ the precise definition of rewilding in the literature has varied.^19^ From “trophic” rewilding^20^ of megafauna to restore top-down trophic interactions and cascades, to passive rewilding, which emphasizes the immediate reduction of human control of an ecosystem, allowing natural regeneration.^17^ Here, we define rewilding broadly as the process of allowing, or facilitating, the restoration of self-sustaining, complex ecosystems that eventually require no or minimum-intervention management.^16^

The socio-political dimensions of rewilding are exemplified in the ongoing discourse about the presence of large carnivores. A spatial linear regression model by Ditmer et al.,^3^ indicated that support for wolf restoration was strongly correlated with Democratic voting patterns in the 2020 US presidential election, with variables such as age, elk hunting, and geographic proximity also influential. Similar research in Washington state and Oregon echoes these findings, showing that political party affiliation strongly predicts attitudes toward wolf management strategies, with Republicans more likely to support wolf control measures.^21^^,^^22^ In Germany, Von Hohenberg and Hager^23^ identified a link between wolf attacks and an increase in far-right voting behaviors.

While there is a growing body of research on the ecological dimensions of rewilding, its socio-economic landscape has received less attention,^12^ despite its potential to disrupt socio-ecological systems.^24^ Notably, there has been limited quantitative study of the social dynamics of rewilding, particularly its political dimensions. In this study, we link preferences for a suite of rewilding interventions to political party affiliation. Specifically, using the unique case study region of the Oder Delta, which spans across Germany and Poland, we employ a discrete choice experiment (DCE) to assess how respondents’ political affiliation affects their willingness to pay (WTP) for different rewilding management alternatives.

In a DCE, respondents choose between alternative scenarios, which reveal the weights they put on different factors.^25^ Discrete choice experiments have been used extensively to investigate public preferences for nature, revealing that the public has WTP for the restoration of natural landscape elements (e.g., Hanley et al.^25^; Senzaki et al.^26^; Tan et al.^27^).

This research highlights the socio-political factors underpinning public attitudes toward rewilding. In Europe, ambitious policy frameworks such as the recently adopted Nature Restoration Regulation (NRR) advocate for restoring biodiversity in Europe, emphasizing the need for transformative change and sustainability in land use.^28^ However, the landscape of environmental policy remains delicate; the advancement of European-wide policies must often navigate a volatile political landscape representing the full spectrum of European political ideologies. Therefore, our research will play a significant role for informing policy by enhancing our understanding of how political affiliation may affect perceptions of rewilding.

### Political landscape, Germany and Poland 2022

**Figure 1:**
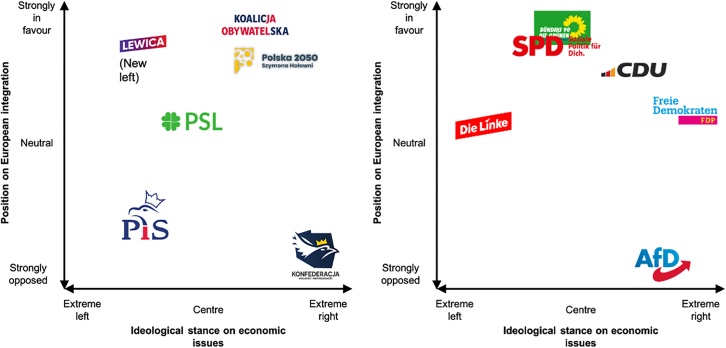
Political map of major Polish (left) and German political parties (right), the position of the parties based on 2019 data from https://chesdata.shinyapps.io/Shiny-CHES/

In August 2022, Poland’s political landscape was characterized by a spectrum of ideologies and policy priorities (Figure 1). The governing Law and Justice (PiS) party, in power since 2015, were ideologically right wing, but to the left economically. By contrast, the Civic Coalition (KO), formed in 2018, unified various parties such as Civic Platform, Modern, and the Greens, advocating for liberal policies and stronger ties with the European Union. The recently founded centrist Poland 2050 party were similarly pro-Europe. The New Left (NL) coalition embraced a left-wing agenda, while the centrist Polish people’s party (PSL) focused on agricultural and rural development. The far-right Confederation (KONF) united right-wing and libertarian groups.

In Germany, the Social Democratic Party (SPD) emerged as the largest party in the 2021 elections. This led to the so-called ruling “traffic light coalition” of the SPD, Free Democratic Party (FDP), and the Greens (Grüne). The SPD and Greens were left of center both ideologically and economically, with the Greens also lobbying for environmental action. By contrast, the libertarian FDP was to the extreme right on economic issues. The three parties were united by a pro-European stance. The second most popular party in the elections, the Christian Democratic Union (CDU), upheld centrist and Christian democratic values and a commitment to European integration. The far-right Alternative for Germany (AfD) promoted an extreme right agenda and euroscepticism. The party’s rhetoric often sparked controversy and debate within German politics.

### Rewilding attributes

**Figure 2:**
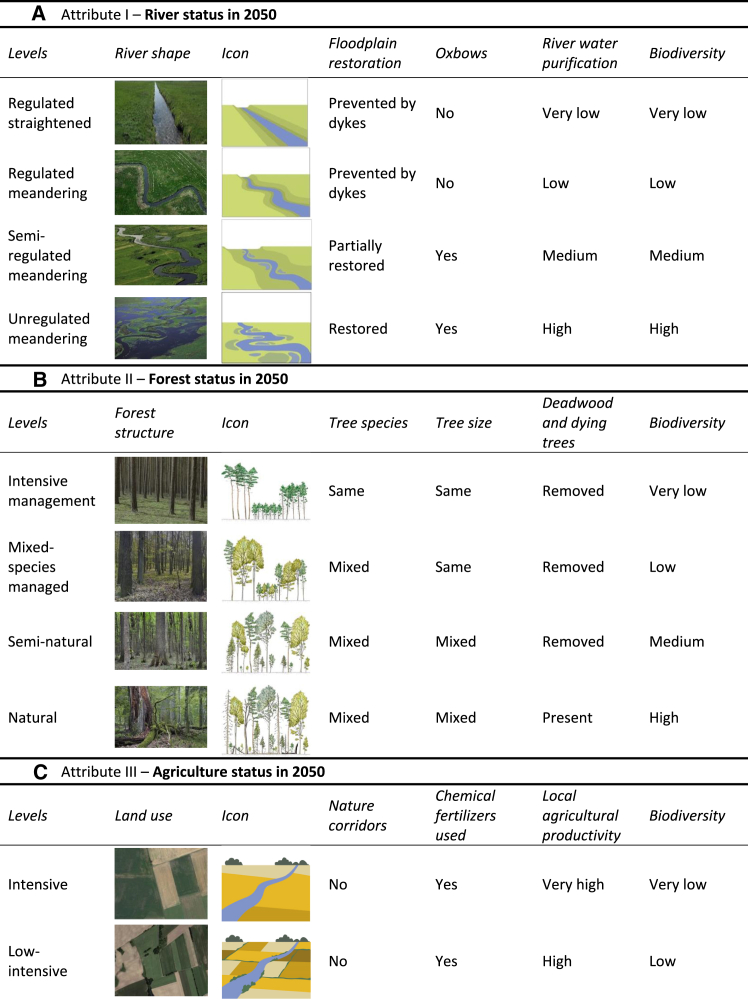

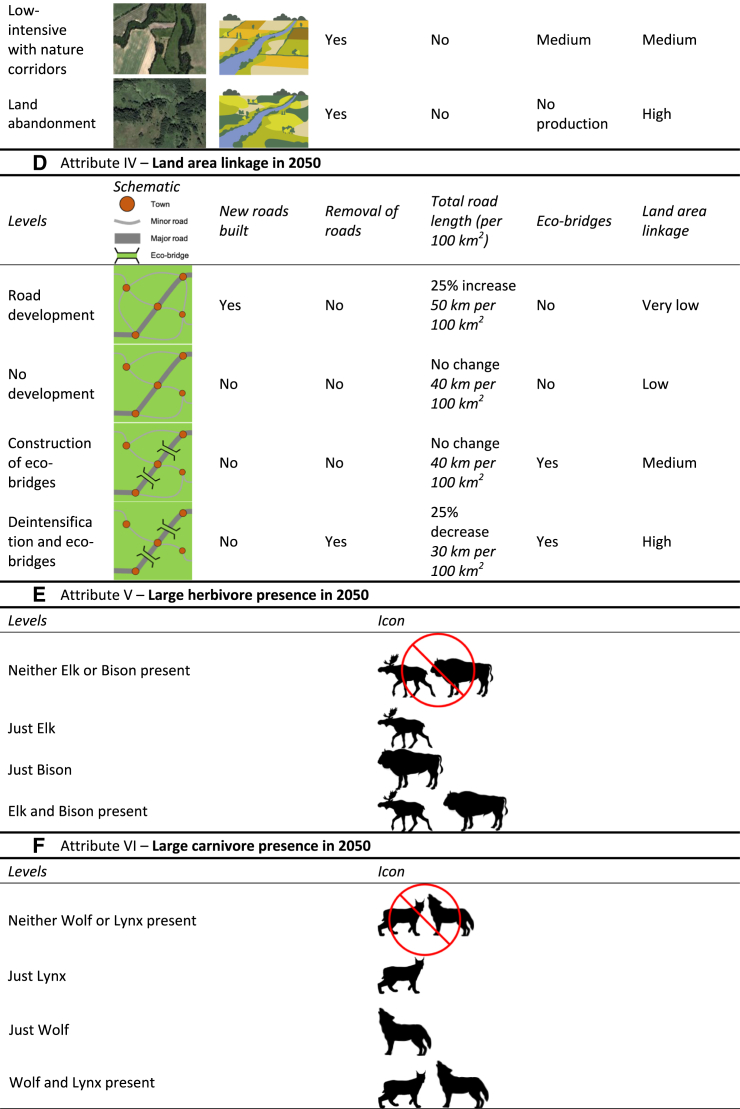
Landscape attributes, levels and their visualization in the discrete choice experiment Photos and icons were shown to respondents in the briefing section of the questionnaire alongside descriptions of the levels, just icons were shown to the respondent during the choice tasks. (A) River status in 2050, ranging from regulated and straightened rivers to unregulated meandering rivers with restored floodplains and high biodiversity. (B) Forest status in 2050, illustrating a gradient from intensively managed monocultures to natural forests with mixed species, deadwood, and high biodiversity. (C) Agricultural land use in 2050, progressing from intensive production with chemical inputs to land abandonment with natural regeneration and enhanced biodiversity. (D) Land area linkage in 2050, showing scenarios from increased road development to eco-bridges and road removal for high landscape connectivity. (E) Presence of large herbivores in 2050, varying from neither species present to both elk and bison being present in the landscape. (F) Presence of large carnivores in 2050, varying from neither species present to both lynx and wolf being present in the landscape.

Attributes in the DCE were designed to act as proxies for the elements of Perino et al.^16^ rewilding framework: large herbivores and large carnivores for trophic complexity; rivers, forests, and agriculture for stochastic disturbances; and connectivity for dispersal (more information on the definition of attributes can be found in Dunn-Capper et al.^29^). We defined interventions as outcomes of changes in management in the Oder Delta that would be observable in 2050, ranging from intensive production (the status quo in this study) to naturalness (fullest extent of rewilding)—reflecting how rewilding interventions exist on a continuum.^30^^,^^31^ These scenarios (Figure 2) were based on the rewilding management plan for the Oder Delta and the expert opinions of researchers and site managers and were designed to be easily achievable through changes in management practices at the site, either through assisted or artificial restoration measures.

## Results

### Influence of voting preferences in Poland

**Figure 3:**
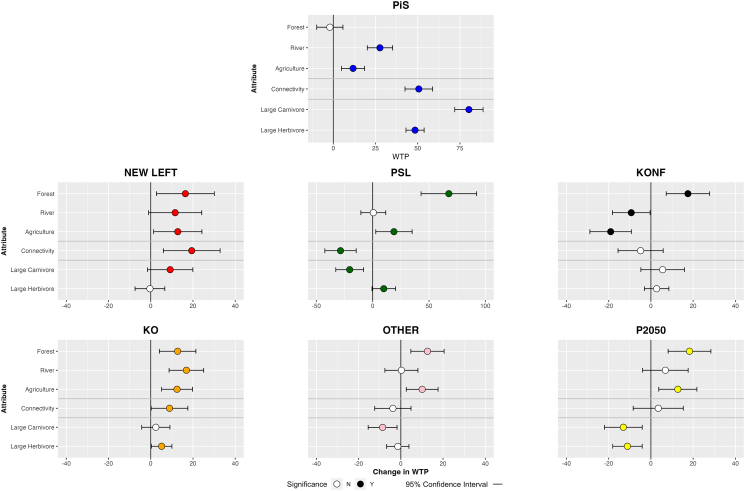
Change in WTP for rewilding attributes for Polish respondents by political affiliation, compared against PiS baseline Top figure presents WTP for the baseline scenario (PIS voter). Bars denote 95% confidence interval, and point fill estimate significance. WTP is in Euros (2022).

The selected estimation results of the mixed logit model for Poland are presented in Figure 3, with more detailed results available in the Supplementary Information (Tables S1 and S2). For the voters of PiS—the party with the largest support at the time of conducting the survey—the reported estimates can be interpreted as marginal mean WTP. For the voters of all the other parties, we estimated the shift in mean WTP, when compared to the voters of PiS (the baseline). As described in the STAR Methods section, the forest, river, agriculture, and connectivity attributes were transformed with a logarithmic function to improve the model’s fit. As such, their marginal WTP values correspond to the improvement from the lowest level (*status quo*) to the subsequent one (see an example of a choice card in SI, Figure S1). For the animal-related attributes, marginal WTP corresponds to any improvement of one level. The large carnivore attribute levels “*just Lynx*” and “*just Wolf*” were merged because we did not observe significant differences in preferences between them in the context of the rewilding program.

Regarding the results for PiS, all attributes related to landscape changes, except for forest, exhibited positive, significant WTP values. The connectivity attribute had the highest WTP (€50.64), followed by river (€27.62) and agriculture (€11.70). The forest attribute showed no significant WTP, indicating a lack of interest among average PiS voters in the rewilding of the forest landscapes. Regarding large carnivores, PiS supporters expressed a WTP of €80.30 for the presence of either a wolf or lynx and €160.60 for the presence of both. For large herbivores, preferences were linear, with a WTP of €48.41 for the presence of elk, €96.82 for the presence of bison, and €145.23 for both species.

For voters of the left-leaning Polish parties, the NL and the KO, a pronounced preference for rewilding interventions was evident. For both parties, we observed a significant shift in WTP for all landscape attributes when compared to the PiS baseline. Additionally, a positive shift was observed for the large animal attributes. NL supporters showed a significantly higher WTP for large carnivores (+€9.20 for the presence of either wolf or lynx), while KO supporters exhibited a significant positive WTP for large herbivores (+€5.14).

Voters of all the other parties exhibited a positive and significant shift in WTP values for rewilding interventions in forest ecosystems. At the same time, the results for other attributes for these parties are less consistent. Notably, we observed some significant negative shifts, indicating a lower WTP for rewilding than the voters of PiS. Voters of KONF had a lower WTP for rewilding of rivers and agriculture, voters of P2050 had a lower WTP for the presence of large carnivores and herbivores, whereas voters of PSL had a lower WTP for increased connectivity and presence of large carnivores.

### Influence of voting preferences in Germany

**Figure 4:**
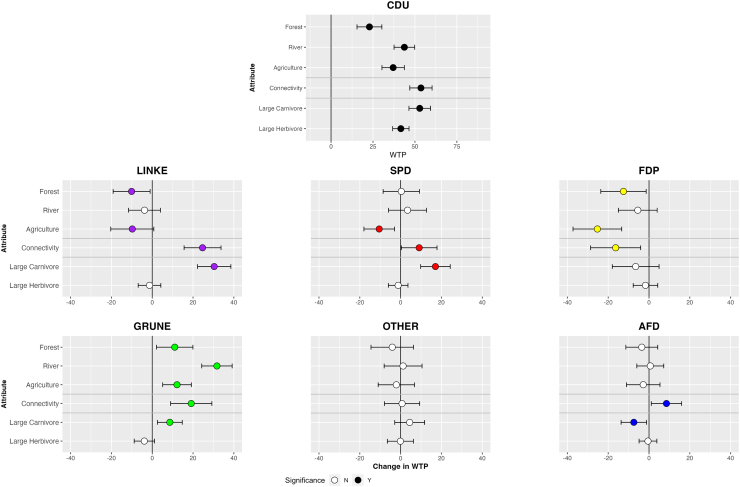
Change in WTP for rewilding attributes for German respondents by political affiliation, compared against CDU baseline Top figure presents WTP for the baseline scenario (CDU voter). Bars denote 95% confidence interval, and point fill estimate significance. WTP is in Euros (2022).

Figure 4 presents the main estimates of the mixed logit model for the German sample. The model follows a specification analogous to that employed for Poland. Detailed results are available in the Supplementary Information (Tables S3 and S4). In Germany, shifts of WTP for each party are calculated relative to the CDU—the party with the largest support at the time of conducting the survey. The mean WTP estimates for CDU voters are relatively similar to those of PiS voters in Poland. These values were positive across all landscape-related attributes, with the highest WTP observed for connectivity (€53.58), followed by rivers (€43.71), agriculture (€37.03), and forests (€22.89). Regarding the presence of large animals, CDU supporters had a value of €52.87 for the presence of either wolf or lynx, €41.60 for the presence of elk, and €83.20 for the presence of bison.

Supporters of the environmental Green party displayed a generally higher WTP for rewilding interventions than CDU voters, with significantly higher values across all attributes except for large herbivores, where no significant change was recorded. On the other hand, the economically right-wing FDP generally exhibited lower WTP for rewilding interventions than the baseline, with significant effects for forest, agriculture, and connectivity attributes. For other parties, results are mixed. The left-wing Die Linke and center-left SPD voters had higher valuations than CDU for enhancing connectivity and increasing the presence of large carnivores. At the same time, they exhibited a significantly lower WTP for rewilding of agricultural systems. Additionally, WTP for rewilding forests was also significantly lower among Die Linke voters. The supporters of the far-right AFD had a significantly higher WTP for connectivity, but lower WTP for the increased presence of large carnivores.

### Importance of voting in explaining preferences

Preference heterogeneity refers to the variation in individuals’ preferences or choices, which can arise due to differences in personal characteristics, experiences, and beliefs. In the context of this study, preference heterogeneity captures how different individuals value various attributes of a rewilding program, such as forest restoration or the introduction of large carnivores, and how their WTP may vary accordingly.^32^

**Figure 5:**
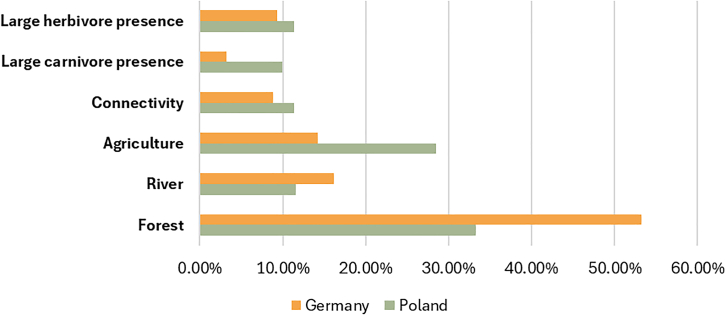
Share of observable preference heterogeneity in willingness-to-pay distribution See Tables S5 and S6 in SM for detailed results.

In the mixed logit model, the variation in WTP distribution can be broken down into the observed and unobserved parts.^32^ For the observed part, variation in WTP is explained by a set of individual-specific variables, including socio-demographic factors and voting preferences (see STAR Methods for further details). This approach aims to capture the deterministic part of the preference heterogeneity (variation in individual choice behavior) for a rewilding program in the Oder Delta and assess the relative importance of voting preferences when compared to other routinely used covariates. The share of the observed preference heterogeneity of WTP is shown in Figure 5. On average, the proportion of explained variation relative to overall variation was very similar in the two countries, at 17.49% in Germany and 17.66% in Poland.

In both countries, the observable covariates explained the substantial part for the rewilding of the forest ecosystem. In Germany, we were able to explain 53% of the total variation for this attribute, whereas for Poland 33% of the total variation. For other attributes, this proportion is considerably lower, averaging around 10%–15% for both countries. The exception is the agriculture attribute in Poland, for which the explained variation amounts to 28% of the total variation. The lowest shares of explained heterogeneity were consistently observed for large carnivore presence, at 3.3% in Germany and 9.9% in Poland. These findings suggest that preference heterogeneity is more easily explained for habitats with higher familiarity and use value (e.g., forests) and remains more cryptic for attributes perceived as unfamiliar or uncertain, such as large carnivores.

In Table 1 the observable preference heterogeneity is further broken down by source. In addition to reporting the relative contribution in percentage terms, we assessed the overall significance of each group of covariates to the log likelihood function using the Wald test.^33^ In both countries, over 60% of the explained variation (65% in Germany and 62% in Poland) is attributable to just two covariates: voting preferences and region of residence, with both contributing almost equally to overall explained preference heterogeneity. Although the explanatory power of these two variables varies across attributes, it is clear that voting preferences exhibited strong relative importance when compared to other socio-demographic factors. The only other covariates that explain a large portion of systematic preference heterogeneity are income in Poland and distance from the Oder Delta in Germany. Among the factors examined, voting preference is the only covariate that exhibits a statistically significant contribution to preference heterogeneity across all rewilding attributes, with a significance level of at least 95% in both Poland and Germany. This finding underscores the relative importance of voting preferences in explaining preference heterogeneity compared to commonly used socio-demographic characteristics.

**Table 1:** Relative contribution of individual-specific covariates to the explained preference heterogeneity in willingness-to-pay distribution (%)

	Distance (log)	Age	Male	City size	Income	Education	Voting	Regions	Sum
**Poland**									

ASC SQ	0.005	3.578∗∗	0.58	4.541∗∗	38.593∗∗∗	1.01	24.227∗∗∗	27.466∗∗∗	100.000∗∗∗
Forest (log)	0.27	4.900∗	2.189	0.136	7.573	4.463∗	57.541∗∗∗	22.928	100.000∗∗∗
River (log)	0.225	0.363	1.117	0.546	12.014	9.707∗∗∗	35.503∗∗∗	40.526∗∗∗	100.000∗∗∗
Agriculture (log)	0.083	6.876∗∗∗	0.143	0.756	4.149	1.679	42.947∗∗∗	43.367∗∗∗	100.000∗∗∗
Connectivity (log)	2.412	2.715∗	13.601∗∗∗	0.828	29.547∗∗∗	3.016∗∗	14.912∗∗∗	32.968∗∗∗	100.000∗∗∗
Large carnivore presence	0.012	0.418	4.120∗∗∗	3.855∗∗∗	55.835∗∗∗	0.749	12.151∗∗∗	22.860∗∗∗	100.000∗∗∗
Large herbivore presence	0.307	0.03	10.106∗∗∗	4.687∗∗∗	34.121∗∗∗	8.467∗∗∗	21.564∗∗∗	20.717∗∗∗	100.000∗∗∗
-Cost (EUR)	0.6	0.546	7.822∗∗∗	2.779∗	28.797∗∗∗	6.330∗∗	19.912∗∗	33.213∗∗∗	100.000∗∗∗
Mean^a^ (non-monetary attributes only)	0.551	2.550∗∗	5.213∗∗∗	1.801∗∗	23.873∗∗∗	4.680∗∗∗	30.770∗∗∗	30.561∗∗∗	100.000∗∗∗

**Germany**									

ASC SQ	3.255	0.248	0.057	5.548∗∗	24.968∗∗∗	4.420∗∗∗	47.393∗∗∗	14.112∗∗	100.000∗∗∗
Forest (log)	31.967∗∗	0.385∗	0.323	2.357∗∗	1.874∗∗∗	0.506∗	4.161∗∗∗	58.426∗∗∗	100.000∗∗∗
River (log)	30.868∗∗	5.297∗∗∗	0.199	0.605	5.326∗∗∗	0.854∗	38.170∗∗∗	18.682∗∗∗	100.000∗∗∗
Agriculture (log)	2.311	0.004	1.115	5.677∗∗	2.691	1.046	41.440∗∗∗	45.717∗∗∗	100.000∗∗∗
Connectivity (log)	0.052	20.422∗∗∗	5.637∗∗∗	1.266	0.996	11.722∗∗∗	42.841∗∗∗	17.063∗∗∗	100.000∗∗∗
Large carnivore presence	17.091	3.639∗∗∗	5.109∗∗∗	0.652	7.829∗∗∗	2.426∗∗	47.709∗∗∗	15.546∗∗∗	100.000∗∗∗
Large herbivore presence	27.094∗	5.832∗∗∗	0.155	7.170∗∗∗	2.404∗∗	0.556	3.376∗∗	53.413∗∗∗	100.000∗∗∗
-Cost (EUR)	21.136	14.008∗∗∗	0.039	7.104∗∗	4.302	7.376∗∗∗	14.858∗∗∗	31.178	100.000∗∗∗
Mean^a^ (non-monetary attributes only)	18.230∗∗∗	5.930∗∗∗	2.089∗∗∗	2.954∗∗∗	3.520∗∗∗	2.852∗∗∗	29.616∗∗∗	34.808∗∗∗	100.000∗∗∗

Notes: ∗∗∗, ∗∗, and ∗ indicate 1%, 5%, and 10% joint significance of individual-specific covariates.

a Mean does not include *ASC SQ* and *Cost* attribute.

### Political map and preferences for rewilding

To summarize preference for rewilding interventions in relation to the political landscape, we map each political party on a two-dimensional plane, indicating its stance on economic issues (left-right) and its position on EU integration (against-in favor). Data on the position of each party was collected from Chapel Hill (https://chesdata.shinyapps.io/Shiny-CHES/). The following metrics were used to explore preferences for rewilding interventions.

(1) Share of the status quo choices: the percentage of choices for which respondents were unwilling to support rewilding interventions at the presented cost levels. This suggests their WTP is below the minimum cost level displayed.
(2) Willingness-to pay for landscape intervention: the mean WTP across interventions for forests, rivers, and agriculture.
(3) Willingness-to pay for connectivity: the WTP for ecological connectivity.
(4) Willingness-to pay for large animals: the mean WTP across interventions for large carnivores and herbivores.

**Figure 6:**
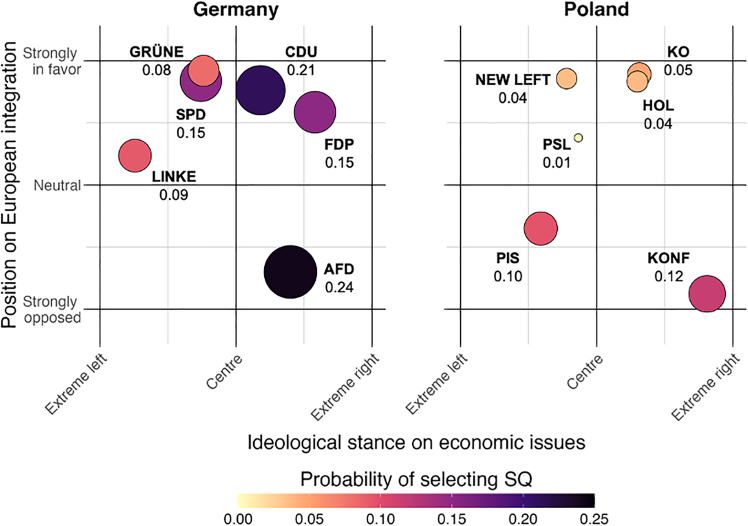
Probability of voters selecting the status quo option (no rewilding)

First, we note that the propensity to choose the status quo was significantly higher in Germany than in Poland: 17.7% vs. 6.7% (Figure 6). In Poland, the share of respondents who chose status quo ranges from 0.5% (PSL) to 11.6% (KONF). By contrast, the levels were substantially higher in Germany, ranging from 8.2% for the Greens to 22.7% for FDP. The largest share of respondents opposing the rewilding program in Poland comes from the right-wing KONF, yet their probability of selecting the status quo was not much larger than that of the Greens, Germany’s most pro-environment party.

In Poland, probability of selecting the status quo aligns with the *y* axis (position on European integration), because parties that supported EU integration had significantly smaller values than those opposed. In Germany, the split was more closely associated with the *x* axis (ideological stance on economic issues), because voters of economically right-wing parties showed a much higher propensity to choose the status quo, indicating the lack of support for rewilding initiatives.

**Figure 7:**
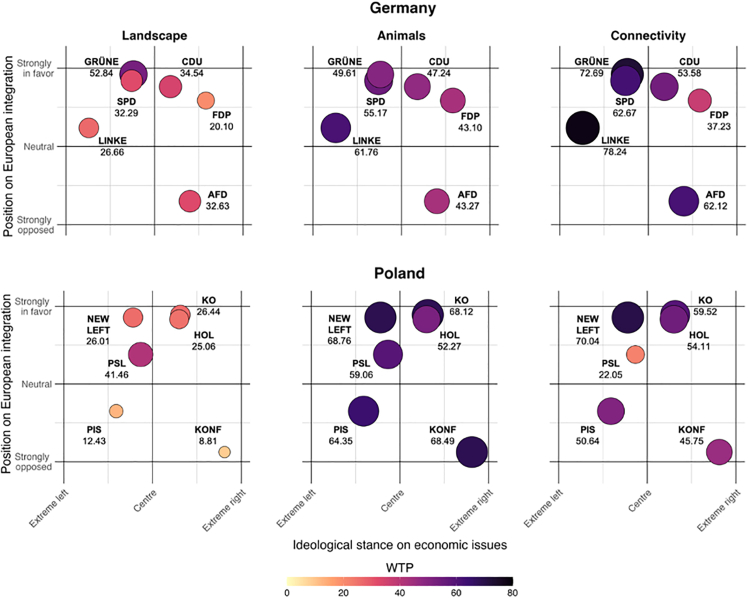
Political party map of Poland and Germany: Willingness to pay (WTP) for rewilding interventions decomposed into three dimensions (landscape, animals, and connectivity) in Euros (2022) See Tables S7 and S8 in SM for detailed results including standard errors.

This pattern largely carries over to the level of WTP for individual rewilding interventions (Figure 7, detailed WTP values are reported in Tables S5 and S6). In Poland, we observe that voters of parties more supportive of EU integration had a systematically higher WTP than those from anti-EU parties. In Germany, voters of more left-leaning parties generally exhibited a higher average WTP. Interestingly, the only notable exception, observed in Poland and to some extent in Germany, is support for large animals. In Poland, there was almost equal support for large animals regardless of party position on the political map, while in Germany, there was also significant support for large animals from AfD voters, comparable to that of left-leaning parties. This finding suggests that, among voters who did not choose the SQ option, support for large animals remains consistently positive and of similar magnitude, regardless of voting preferences. However, when assessing overall support for the rewilding program—which includes large animals—it is essential to account for the substantial variation in SQ option (no rewilding program) selection across voters of different political parties.

## Discussion

Applying a DCE approach to assess public WTP for rewilding interventions in the Oder Delta, we explore the intricate relationship between political affiliation and support for rewilding. The findings from both Germany and Poland reveal politics as a significant determinant of preference for rewilding interventions, with supporters of left-leaning parties generally exhibiting a higher WTP for such initiatives. These insights hold considerable relevance at the policy level as rewilding gains traction as a strategy for ecological restoration.

Our study shows that political affiliation is correlated with degree of support for rewilding measured by WTP but also for the probability of supporting a rewilding program (i.e., 1—probability of selecting the status quo). In both countries, political orientation emerges as a major predictor of rewilding preferences.

In addition to voting, distance and region are two other key factors in predicting preference, particularly in Germany. This finding resonates with the concept of distance-decay, where WTP tends to diminish as the respondent’s distance from the site of interest increases.^34^^,^^35^ This trend is a well-established finding in the valuation literature.^36^ Often, distance-decay is linked with the concept of a substitution effect, wherein the number of available substitutes to the valued area increases with distance from the original site.^37^

Over time, environmentalism has become more closely aligned with leftist political ideologies.^1^ Yet, WTP for rewilding interventions is not uniform across all left-leaning parties in our study. In Germany, Green Party voters demonstrated increased WTP for all rewilding attributes, excluding large herbivores, when compared to the CDU baseline. However, our study highlights a more nuanced position of (far-left) Die Linke and (centre-left) SPD supporters, who exhibit selective WTP for funding rewilding initiatives. This finding may reflect an ideological tension within certain leftist parties, who must reconcile aspirations for economic growth—aimed at improving the conditions for the working-class—with emerging demands for environmental stewardship that may conflict with economic initiatives.^1^^,^^38^ The Green Party in Germany has a complicated relationship with the other left-wing parties,^39^ highlighting the complex socio-economic priorities intertwined with environmental advocacy within left-leaning political groups. This is illustrated in Germany by the recent fracturing of Die Linke, with several former leading members breaking away to form a new party, with a heavy focus on the government’s current climate policy and “culture war” topics.^40^^,^^41^ They are especially critical of eco-activism, instead prioritizing industrial and technological solutions to the climate crisis. By contrast, the regrouping process of Die Linke has triggered a shift toward green policies.^41^

Conversely, our study showed a general lower willingness-to-pay for rewilding initiatives among right-leaning and far-right party supporters. This resistance, particularly stark within Poland’s KONF and Germany’s FDP, may be attributed to a broader skepticism toward environmental policies, which often intersect with nationalist and economic-first agendas. Nonetheless, certain rewilding attributes are valued highly among right-leaning parties, suggesting a more complex stance toward environmental conservation. Understanding these divergent views is essential for fostering coexistence between humans and wildlife.

The politicization of the wolf’s return in Europe exemplifies this complex interplay between political affiliation and environmental attitudes. Conservation efforts, particularly the reintegration of large predators into human-dominated landscapes, can reignite historical cultural disputes and tensions.^42^ While many consider large carnivores to be charismatic, valuable species to be protected,^43^^,^^44^ others view their presence as a threat to their safety and livelihoods.^43^ This may be especially pronounced in rural communities where the reintroduction of species such as the wolf represents a perceived threat to traditional lifestyles.^42^^,^^45^^,^^46^ Such conflicts may be driven by different world views of participants, caused by differences in background and belief,^47^ and are difficult to reconcile. Solutions must take into account stakeholder values and perceptions.^47^

Prior studies have shown a link between political affiliation and attitude toward the wolf.^3^^,^^21^ For example, van Eeden et al.,^21^ found voters self-identifying as Democrats were more likely to hold positive attitudes to wolf than supporters of other political parties, while Republicans showed greater support for wolf elimination. In Spain, right-wing campaigns aim to legalize wolf hunting again,^48^ while in Germany, the high-profile killing of the President of the European Commission’s family pony caused popular outcry.^49^ Von Hohenberg and Hager^23^ link increased far-right voting in Germany to wolf attacks; this finding holds for our study, in which AfD voters were the only group with lower WTP for large carnivore return compared to the CDU baseline.

However, this negative framing of the wolf contrasts with certain far-right views that celebrate nature. For instance, in Germany, in some far-right imaginaries, Germans and “the land” are connected, with the “German forest” a powerful symbol of Germandom.^50^ Interestingly, in our study we find no difference in WTP between the AfD and CDU for rewilding forest landscapes. In Poland, the environment is a relatively new site of political discourse, and environmentalism is much more grassroots.^51^ Similarly to in Germany, right-wing parties emphasize the importance of natural heritage and maintaining mutual complementation with natural land,^51^ which may explain the positive change in WTP for forests among KONF voters.

Understanding the relationship between political orientation and rewilding preferences is critical for effective environmental decision-making, especially in the context of growing ideological polarization surrounding environmental issues.^52^ Rewilding remains a contentious topic among stakeholders, often intersecting with broader disputes between conservation goals and competing land-use interests.^24^ Redpath et al.,^53^ highlight the importance of distinguishing between conflicts directly involving wildlife and deeper, value-based conflicts among people themselves.^47^^,^^53^ Our findings illustrate how rewilding preferences are shaped by political identity. As political parties increasingly view their oppositions not just as rivals, but existential threats,^54^ perceived political bias in conservation efforts may fuel distrust, escalate community-level conflicts, and reinforce partisan group identities.^21^ A deeper understanding of how political identities shape environmental preferences is therefore essential for crafting inclusive, socially sustainable conservation strategies and for mitigating human-human conflicts.

In the context of rewilding, our study reveals widespread support for rewilding from across the political spectrum; however, there is potential for increasing political polarization in the future as rewilding becomes more prominent in Europe. Brulle et al.,^55^ show that the public’s environmental views are shaped more by political messaging than scientific communication. This highlights the crucial role that politicians play in influencing public perceptions of the environment. Not only does political affiliation influence support for environmental protection programs, but also for environment-friendly behaviors and responses to campaigns that promote such behaviors.^2^

This influence can also work in the opposite direction, as politicians have the potential to sway voters’ preference toward more negative positions regarding the environment. For example, we see in Poland that all parties have a significant positive shift in WTP for rewilding in forests compared to PiS. One potential reason for this could be the strong ties that PiS had with State Forests—an institution managing a vast portion of state-owned assets—while they were in power. During its tenure, PiS unequivocally supported the intensification of forest management, including increasing logging in certain areas, such as the Białowieża Forest—a biodiversity-rich, ancient forest. This issue sparked a major international scandal and intervention by EU institutions.^56^ During PiS’s tenure, topics related to forest management and the influence of EU institutions were regularly highlighted in government-aligned media (e.g., Cukiernik^57^). These narratives often framed EU interventions as attempts to interfere in Poland’s internal affairs and could have contributed to the negative perception of rewilding in forests among PiS voters in our study.

The findings from this study are particularly significant to the ongoing debate on the NRR in Europe.^58^ Our results suggest that rewilding interventions—particularly those involving the restoration of large animals—may be politically desirable at the national level. A recent Rewilding Europe report highlights the potential of species reintroductions to contribute to the objectives of the NRR.^59^ For example, restoring natural grazing regimes by reintroducing large herbivores, for which elk and bison are two of the main species in Europe. Restoring large herbivores may not only be desirable to the general public from a holistic standpoint, but also benefit society through wider ecosystem functioning.^60^ Similarly, the reintroduction of large carnivore can help restore trophic chains.^59^ At the time of study, lynx and bison were absent from the German side of the Oder Delta. Our results suggest their reintroduction in this region may be desirable at the national level. However, these efforts must account for local people, for whom preferences often differ.^29^ Political and legislative tools, such as subsidies, could help facilitate reintroductions and mitigate associated biodiversity impacts.^47^

Poland has become explicit opponents of the law, with leader Donald Tusk of KO arguing that Poland would protect nature “without European coercion”,^61^ which has been met with strong opposition from their Left-wing coalition partners. While this study shows strong preference for rewilding interventions among Polish respondents, and especially KO supporters, there exists a danger of this being eroded by strong public political messaging.^2^ This underscores the critical importance for science to continue to inform politicians across the political spectrum of the potential ecological and economic benefits of rewilding.

### Limitations of the study

While DCEs offer a powerful tool for eliciting preferences for complex and multidimensional environmental interventions, such as rewilding, they are not without shortcomings. The use of hypothetical scenarios, the assumption of stable and compensatory preferences, and the cognitive demands placed on respondents may influence results.^62^^,^^63^ Nonetheless, DCEs remain one of the most robust stated preference methods available for estimating the non-market value of environmental changes, especially when real-world market data are absent.^62^^,^^64^

Our choice of a DCE was motivated by its ability to disentangle preferences for specific components of rewilding, which are inherently multidimensional and often contested. In the context of the Oder Delta, where multiple and sometimes conflicting visions of nature coexist, a DCE allows for an exploration of the trade-offs individuals are willing to make between various rewilding interventions, as well as between ecological goals and socio-economic implications. Importantly, it also enables the estimation of WTP, which is essential for informing policy decisions grounded in natural capital accounting and cost-benefit analysis.^62^^,^^65^

Despite its strengths, the DCE approach also implies simplifications. Respondents are required to make choices based on limited and stylized descriptions of interventions, which may not capture the full complexity or uncertainty associated with ecological processes or local realities. Moreover, while we attempted to ensure relevance and comprehensibility through cognitive testing and piloting, there remains a risk that respondents may interpret attributes differently or rely on heuristics.^66^

We have conducted a latent class analysis of the DCE data to try to identify potential groups of respondents that may behave differently than assumed by the random utility-based choice models. We found that there are respondents who ignore a cost attribute, or for whom the cost effect is positive rather than negative. As a robustness check, we have therefore re-estimated the models on the subsample excluding those individuals (based on the self-reported indicator of the cost non-attendance). In general, the results were robust, although, as expected, the WTPs were lower in the subsample, especially for the case of Germany. In the case of Poland, the largest differences were observed for voters of PSL and KONF parties—the outcome that can be explained by their low sample sizes. As such, we recommend caution when interpreting the results for these parties. In the case of Germany, the differences in the estimated WTP between the samples were larger, although the relative positioning of the parties in terms of WTP was rather robust.

Finally, preferences captured through DCEs represent stated rather than revealed behavior, and as such, results should be interpreted with caution when extrapolating to real-world decision-making contexts.^67^

## Resource availability

### Lead contact

Further information and requests for resources should be directed to and will be fulfilled by the lead contact, Marek Giergiczny (m.giergiczny@uw.edu.pl).

### Materials availability

The study did not generate new unique reagents.

### Data and code availability

The data used in this study was collected from online survey responses; as part of the survey each respondent completed 12 choice tasks. Anonymized choice experiment data and results may be found at: https://doi.org/10.5281/zenodo.14864681.

• Data: All data used in this study, including cleaned and anonymized datasets for Germany (DE_data.mat) and Poland (PL_data.mat), are available in the Zenodo archive https://doi.org/10.5281/zenodo.14864682 under the DE/and PL/directories.
• Code: The full set of MATLAB scripts used for model estimation and table generation—including PL_model_estimation.m, DE_model_estimation.m, and associated code for output tables—is available in the same repository. Estimation routines are organized into the Estimation Package/folder with separate subfolders for MNL and MXL models.
• Other materials: The archive also contains model outputs (∗_model_results.mat), code-generated summary tables (e.g., Table_A1_A2.m, Table_A6.m, etc.), and supplementary utilities (in Tools/, circlem/, mmx_package/, and xls_templates/) used to support estimation and performance optimization.

## Acknowledgments

This work was supported by the project TERRANOVA the European Landscape Learning Initiative, which has received funding from the 10.13039/501100007601European Union’s Horizon 2020 Research and Innovation program under grant agreement no 813904. R.D.-C., N.F., and H.M.P., gratefully acknowledge the support of iDiv funded by the German Research Foundation (DFG- FZT 118, 202548818), M.G. acknowledges the financial support from the National Science Centre in Poland (2021/43/B/HS4/03371) and the iDiv Flexpool funds. We would like to thank Rewilding Oder Delta e.V. for their help in the survey design, and Fabian Marder for his help with data collection.

## Author contributions

H.M.P., R.D.-C., and M.G. conceived the idea for the research; R.D.-C. and M.G. designed the methodology, supported by N.F. and H.M.P.; M.G. and W.B. led the data analysis; R.D.-C. and M.G. led the writing of the manuscript, supported by H.M.P., N.F., and W.B. All authors contributed critically to the drafts and gave final approval for publication.

## Declaration of interests

The authors declare no competing interests.

## STAR★Methods

### Key resources table

**Table:** 

REAGENT or RESOURCE	SOURCE	IDENTIFIER
**Deposited data**		

Anonymized survey data	This paper	https://doi.org/10.5281/zenodo.14864681
Statistical analysis and results	This paper	https://doi.org/10.5281/zenodo.14864681

### Experimental model and study participant details

To determine respondent WTP for rewilding interventions in the Oder Delta, we conducted a DCE. The survey was conducted online (CAWI mode) in both Germany and Poland, with respondents selected by professional online survey companies. The surveys for both countries were conducted in August and September 2022, resulting in a total of 1,005 completed surveys for Germany and 1,066 for Poland after data cleaning. The samples for both countries were representative with respect to: age, gender, region and municipality size.

Given the nature of this study, and in compliance with university guidelines at the time, ethical approval for this study was granted by the lead supervisor responsible for the project. Care was taken to ensure that all data collected was anonymized, that no personal or sensitive data were collected and all participants were over the age of 18. Individual respondents were only identifiable in the results through a unique numerical ID. Participants took part in the survey voluntarily and by submitting the survey gave consent for their anonymized data to be used. They could back out at any time before submitting.

#### Study site

The Oder Delta, spanning approximately 450,000 ha across the Germany-Poland border, lies along the Baltic coast and includes the 70,000 ha Szczecin Lagoon. The area features a rich mosaic of landscapes including riparian and swamp forests, deciduous and coniferous forests, peatlands, standing- and flowing waters, dunes, and heathlands. Approximately 40% of the total terrestrial Oder Delta area is part of the European Natura2000 network, and the region is surrounded by heterogeneous landscapes of forests, rivers, and wetlands, making it suitable for the comeback of natural wildlife.

Like many European landscapes,^68^ the Oder Delta has been shaped significantly by human activity over the past centuries. In particular, the creation of dams and dikes increased the amount of land available for agriculture and forestry, which are now major land uses in the region. Nowadays, the Oder Delta is popular with tourists, offering activities such as birdwatching, hiking, wildlife observation, and swimming in the lakes and the Baltic Sea.

At the time of study, rewilding interventions were already ongoing in the Oder Delta region, spearheaded by the Rewilding Oder Delta e.V. These were largely focused on freshwater systems, for example restocking important fish species in the Szczecin Lagoon. In terrestrial areas, plans included allowing and supporting the comeback of elk and bison, diversifying forest structures, and restoring hydrological regimes by removing obsolete dams.

### Method details

#### Study design

The full details of the study design and implementation are reported in Dunn-Capper et al.,^29^ here we only present the main elements of the study.

To account for the uncertainty implicit in rewilding outcomes, the levels of the landscape attributes (forests, rivers, and agriculture) included elements of stochastic disturbances. For rivers, flooding regimes were restored; for forests, deadwood was left on the forest floor, and in agricultural landscapes all management of the land was stopped, fully allowing natural processes to take over. This was described to the respondents before the choice tasks, and also care was taken to ensure these disturbances were well represented in the icons.

The icons depicting the attribute levels were designed to be understood by respondents with limited prior knowledge of the ecological concepts underpinning the framework. In addition to icons and photographs all levels were also explained using written descriptions with language understandable to the general public. Cost was defined as a new annual obligatory tax paid by all citizens in the respondents’ country for the foreseeable future. An example of a choice card is presented in Supplementary Information (Figure S1). Each respondent saw 12 choice cards, and were asked to select the best option out of two program alternatives and the status quo (SQ).

#### Data collection

The primary test for Germany and Poland was conducted in August and September 2022, resulting in a total of 1,657 completed surveys collected for Germany and 1,514 for Poland. Of these, the survey company collected approximately 500 respondents from each country residing within or neighboring the national state containing the Oder Delta region (e.g., Mecklenburg-Vorpommern in Germany and the West Pomerania Province in Poland). The aim of this was to try to collect respondents living locally to the Oder Delta.

Quality control questions were included in the survey to ensure respondents were reading the questions and responding accurately. The collected data were then cleaned to remove respondents identified as speeders (those below 50% of the median time), those that failed the quality control questions, or those identified to have given protest responses. After cleaning, there were 1,005 completed surveys for Germany and 1,066 for Poland.

#### Model specification

We applied an MXL^69^ to analyze the data from the DCE. The model is rooted in random utility theory,^70^ which assumes that an individual’s (*n*) preference can be decomposed into a deterministic component $\left(V_{itn}\right)$ and an unobservable, stochastic component $\left(\varepsilon_{itn}\right)$. This leads to the usual formulation of the utility that an individual derives from choosing alternative *i* at the choice occasion *t*,

(Equation 1) $$U_{itn}=V_{itn}+\varepsilon_{itn}$$

In this study, we specify the deterministic component of the utility as

(Equation 2) $$V_{itn}=\alpha_{n}\left(\beta_{n0}SQ_{itn}+\beta_{n1}{\log\;L}_{itn}+\beta_{n2}LC_{itn}+\beta_{n3}LH_{itn}-\mathrm{Cos}t_{itn}\right)\text{.}$$

Here, the model in Equation 2 is specified in the WTP-space,^71^ so that $\alpha_{n}$ coefficient represents the marginal utility of money (confounded with the scale), whereas the β coefficients can be interpreted directly in monetary terms as a willingness to pay (WTP).

$SQ_{itn}$ is a dummy variable, equal to 1 for the status quo (SQ) alternative, and $\beta_{n0}$ is therefore an SQ alternative specific constant (ASC). ${\log\;L}_{itn}$ is a vector of landscape change attributes, namely forest, river, agriculture, and connectivity. These attributes are categorical variables, however are treated as continuous for the purpose of the model (This was done to limit the number of coefficients in the model, as they increase extremely quickly when using an MXL model with a full correlation matrix and interactions with socio-demographic variables). To account for decreasing marginal utility, these attributes were log-transformed. $LC_{itn}$ and $LH_{itn}$ correspond to the presence of large carnivores and herbivores, respectively, and are treated as continuous variables. Finally, $\mathrm{Cos}t_{itn}$ represents the monetary attribute of the annual increase in taxes.

We assume that each respondent in the sample has a separate set of parameters, which is highlighted by them having a lower index *n*. As a set of coefficients for each individual cannot be directly estimated, we instead decompose them into observable and stochastic parts,

(Equation 3) $$\left\{ \begin{array}{c} \beta_{nj}=\mu_{j}+\lambda_{j}X_{n}+\zeta_{nj}\\ \alpha_{n}=\exp\left(\pi +\gamma X_{n}+\eta_{n}\right)\text{.} \end{array}\right.$$

We assume that coefficients that can be interpreted as WTP ($\beta_{nj}$), follow a normal distribution, with the mean of the distribution being additionally explained by the set of individual-specific variables ($X_{n}$), which includes voting preferences. The marginal utility of money ($\alpha_{n}$) is assumed to follow a log-normal distribution.

Due to the unobserved stochastic terms in Equation 3, the likelihood function does not have an analytical form, but instead is a multidimensional integral

(Equation 4) $$L_{n}=\int \prod_{t}P\left(y_{tn}|\zeta_{n},\eta_{n}\right)f\left(\zeta_{n},\eta_{n}|\Omega \right)d\zeta_{n}d\eta_{n}\text{.}$$

In Equation 4, $f\left(\zeta_{n},\eta_{n}|\Omega \right)$ is a density function of the multivariate normal distribution, with the mean zero, and covariance matrix, Ω, being fully estimated. $P\left(y_{tn}|\zeta_{n},\eta_{n}\right)$ denotes a choice probability with $y_{tn}$ being a vector of zeros and ones, with one indicating a chosen alternative. Assuming Gumbel-distributed error terms, $\varepsilon_{itn}$, in Equation 1, leads to a well-known multinomial logit choice probability,

(Equation 5) $$P\left(y_{tn}|\zeta_{n},\eta_{n}\right)=\prod_{j}{\frac{\exp\left(V_{jtn}\right)}{\sum_{l}\exp\left(V_{ltn}\right)}}^{y_{jtn}}\text{.}$$

We estimated the model using the Maximum Simulated Likelihood approach with 2,000 scrambled Sobol draws^72^ to approximate the integral in Equation 4.

### Quantification and statistical analysis

#### Model interpretation

To interpret the MXL results, we consider two elements. First, the model’s coefficients in Equation 2 can be interpreted in monetary terms. For instance, $\beta_{n3}$ can be interpreted as an individual’s WTP for a unit increase in the presence of large herbivores. This interpretation becomes slightly more complex for the log-transformed attributes, illustrated by,

(Equation 6) $$WTP_{n1}=\frac{\beta_{n1}}{L_{itn}}\text{.}$$

When the attribute equals 1, we obtain $WTP_{n1}=\beta_{n1}$, which is analogous to the other attributes, but this equivalence does not hold for all levels of the attribute.

Second, we evaluate importance of individual-specific variables by decomposing the variation of random parameters presented in Equation 3. For the *k*-th covariate, we calculate the share of explained variance as

(Equation 7) $$Var_{jk}=\frac{\lambda_{jk}^{2}var\left(X_{k}\right)}{var\left(\lambda_{j}X\right)+var\left(\zeta_{j}\right)}$$

Here, $X_{k}$ denotes the vector of all observations for the *k*-th variable in the sample. Therefore, variance is calculated based on the variation between different individuals.
