# Supplementary material for: The political dimensions of rewilding preference
Source: iScience. 2025 Sep 3;28(9):113349. doi: 10.1016/j.isci.2025.113349 (PMC12496218; doi:10.1016/j.isci.2025.113349)
Supplement: Document S1. Figure S1 and Tables S1–S6 [file mmc1.pdf]

iScience, Volume *28*

Supplemental information

The political dimensions of rewilding preference

Marek Giergiczny, Rowan Dunn-Capper, Wiktor Budziński, Nestor Fernandez, and Henrique M. Pereira

Figure S1. Example choice card

	Projected, 2050	Program A, 2050	Program B, 2050
**Rivers**	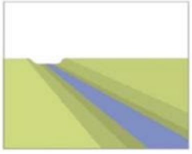	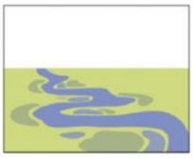	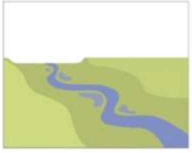
**Forests**	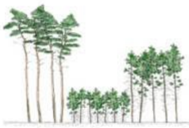	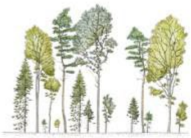	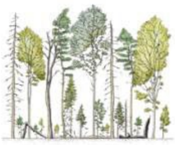
**Land area linkage**	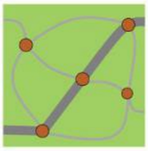	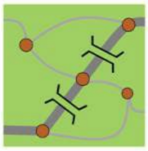	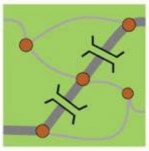
**Large herbivores**	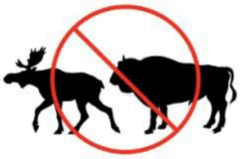	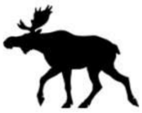	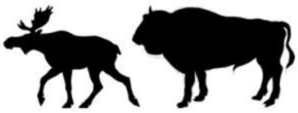
**Yearly tax increase (EUR)**	**0**	**20**	**80**

Table S1. Results of the MXL model: the case of Poland. Part 1: Voting behavior.

	Random parameters		Voting: base level is PIS						
	**Mean**	**Std. dev.**	**Wouldn't vote**	**KO**	**P2050**	**NEW LEFT**	**PSL**	**KONF**	**Other**
**ASC SQ**	-124.471 ***	75.346 ***	14.39	-15.656 **	-15.777 *	-16.413	-63.674 ***	10.116	4.935
	(8.962)	(4.823)	(9.843)	(7.116)	(8.938)	(10.227)	(17.754)	(9.949)	(7.109)
**Forest (log)**	-2.034	16.157 ***	8.961 *	12.721 ***	18.285 ***	16.394 **	67.634 ***	17.526 ***	12.617 ***
	(3.957)	(1.842)	(5.180)	(4.414)	(5.169)	(6.991)	(12.594)	(5.250)	(4.019)
**River (log)**	27.62 ***	35.029 ***	0.506	16.871 ***	6.853	11.55 *	0.57	-9.305 **	0.261
	(3.820)	(1.715)	(5.119)	(4.184)	(5.504)	(6.424)	(5.640)	(4.548)	(3.991)
**Agriculture (log)**	11.703 ***	22.441 ***	-11.655 **	12.443 ***	12.756 ***	12.789 **	18.874 **	-19.067 ***	10.095 ***
	(3.497)	(1.574)	(5.390)	(3.726)	(4.609)	(5.836)	(8.228)	(5.017)	(3.844)
**Connectivity (log)**	50.639 ***	55.478 ***	4.058	8.882 **	3.469	19.401 ***	-28.591 ***	-4.886	-3.775
	(4.166)	(2.229)	(6.336)	(4.431)	(6.055)	(6.860)	(7.099)	(5.454)	(4.388)
**Large carnivore presence**	80.299 ***	61.018 ***	-8.113	2.401	-13.06 ***	9.195 *	-20.433 ***	5.555	-8.636 **
	(4.276)	(2.433)	(5.149)	(3.411)	(4.560)	(5.486)	(6.292)	(5.266)	(3.492)
**Large herbivore presence**	48.406 ***	31.269 ***	-5.216	5.141 **	-11.115 ***	-0.394	9.843 *	2.711	-1.441
	(2.740)	(1.454)	(3.355)	(2.485)	(3.562)	(3.594)	(5.342)	(2.959)	(2.696)
**-Cost (EUR)**	0.028 ***	1.291 ***	-0.195	-0.106	0.216	0.186	1.342 ***	0.131	0.07
	(0.003)	(0.064)	(0.187)	(0.139)	(0.168)	(0.208)	(0.480)	(0.231)	(0.138)

Notes:

[1] ***, **, and * indicate 1%, 5%, and 10% significance levels, respectively.

[2] The model was estimated in WTP-space and then rescaled, so that the column "Mean" would represent the average WTP for the voter of PiS (base level) with all other characteristics at the mean level. The interaction effects with other political parties represent a deviation from the average WTP of PiS voter.

[3] Figure 2 in the main text is based on the results reported in this table.

[4] All coefficients, except for *Cost*, can be interpreted in monetary terms (EUR). We assumed 1 EUR ≈ 4.75 PLN.

[5] *Cost*'s random parameter has a log-normal distribution. In column "Mean" we report a median of this distribution, whereas in column "Std. dev." we report the standard deviation of the underlying normal distribution. Interaction terms refer to the change in the mean of the underlying normal distribution as per formula (3).

Table S2. Results of the MXL model: the case of Poland. Part 2: Other effects.

								Regions: base level is Mazowieckie			
	Distance (log)	Age	Male	City size	Income	Income missing	Education	Dolnoslaskie	Kujawsko - pomorskie	Lubelskie	Lubuskie
**ASC SQ**	0.191	33.508 **	-4.031	3.173 **	-6.379 ***	12.156 *	5.438	18.36 *	28.073 **	51.748 ***	15.146
	(4.499)	(15.469)	(4.758)	(1.371)	(1.648)	(6.880)	(4.392)	(9.626)	(14.220)	(9.163)	(15.211)
**Forest (log)**	0.589	-16.775 *	-3.351	-0.235	0.962	-6.391	4.89 *	-8.618	-7.808	-1.991	4.552
	(2.393)	(9.859)	(2.947)	(0.777)	(0.965)	(4.148)	(2.680)	(5.244)	(11.561)	(6.382)	(12.181)
**River (log)**	-0.595	-5.055	-2.651	0.521	1.735 **	-0.951	7.985 ***	7.599	4.365	2.825	32.283 ***
	(2.430)	(8.941)	(2.502)	(0.680)	(0.838)	(3.585)	(2.593)	(5.551)	(10.726)	(5.705)	(7.963)
**Agriculture (log)**	-0.394	-24.04 ***	-1.035	-0.67	0.279	-8.991 **	3.628	15.06 ***	-8.504	11.351 **	48.688 ***
	(2.469)	(8.853)	(2.652)	(0.830)	(0.835)	(4.339)	(2.589)	(5.019)	(9.283)	(5.063)	(7.699)
**Connectivity (log)**	-3.011	-21.363 *	-14.29 ***	0.992	4.117 ***	-6.688 *	6.876 **	20.723 ***	42.059 ***	20.276 ***	22.949 ***
	(2.520)	(11.314)	(2.877)	(0.742)	(0.936)	(3.876)	(2.838)	(6.334)	(11.012)	(6.153)	(8.403)
**Large carnivore presence**	-0.213	-8.571	-8.041 ***	2.187 ***	5.906 ***	6.832 *	-3.504	11.519 ***	28.235 ***	8.546 *	16.275 **
	(2.065)	(7.714)	(2.199)	(0.698)	(0.780)	(3.784)	(2.324)	(4.471)	(7.284)	(4.409)	(6.591)
**Large herbivore presence**	-0.6	-1.261	-6.872 ***	1.316 ***	2.526 ***	2.314	-6.427 ***	2.714	7.156	-5.288	22.539 ***
	(1.199)	(5.907)	(1.673)	(0.461)	(0.578)	(2.376)	(1.590)	(3.508)	(5.532)	(3.291)	(5.874)
**-Cost (EUR)**	-0.035	0.224	0.253 ***	-0.042 *	-0.097 ***	-0.095	0.233 **	-0.017	-0.679 ***	0.298	-0.235
	(0.050)	(0.324)	(0.086)	(0.025)	(0.018)	(0.137)	(0.098)	(0.171)	(0.223)	(0.232)	(0.322)

Regions (continued): base level is Mazowieckie

	Lodzkie	Malopolskie	Opolskie	Podkarpackie	Podlaskie	Pomorskie	Slaskie	Swietokrzyskie	Warminsko - Mazurskie	Wielkopolskie	Zachodnio - pomorskie
**ASC SQ**	14.501	20.018 *	33.147 ***	-2.481	4.975	6.117	13.05	-1.211	11.276	40.187 ***	5.355
	(12.164)	(10.358)	(10.107)	(12.196)	(15.247)	(11.071)	(9.218)	(14.762)	(11.836)	(10.365)	(16.908)
**Forest (log)**	-1.574	-1.329	-14.063 *	-2.863	-5.951	-2.612	-3.84	-17.646	-6.657	-15.849 ***	-12.751
	(7.394)	(6.200)	(7.461)	(7.242)	(9.010)	(5.865)	(5.017)	(12.637)	(8.219)	(6.066)	(8.861)
**River (log)**	7.24	-5.026	18.316 ***	9.68	-7.689	1.29	-0.564	18.681	0.348	13.872 ***	-3.952
	(6.508)	(6.262)	(6.204)	(6.709)	(7.276)	(5.416)	(4.969)	(11.895)	(6.779)	(4.907)	(11.112)
**Agriculture (log)**	5.328	4.681	5.056	9.297	1.306	11.445 *	11.548 **	18.728 **	6.447	16.185 ***	-0.488
	(6.625)	(5.202)	(6.340)	(6.914)	(8.570)	(5.945)	(4.684)	(9.270)	(6.784)	(6.243)	(9.203)
**Connectivity (log)**	25.793 ***	3.224	25.26 ***	4.962	11.043	6.029	20.92 ***	-1.683	24.359 ***	11.197 *	-0.173
	(6.760)	(7.467)	(8.486)	(8.433)	(9.420)	(6.513)	(5.893)	(11.108)	(7.462)	(5.928)	(9.613)
**Large carnivore presence**	1.283	-10.556 *	8.986	-8.728	-5.045	-11.131 **	4.558	-4.435	-9.396	-3.842	10.837
	(5.869)	(6.128)	(5.845)	(7.282)	(6.927)	(4.472)	(4.722)	(11.244)	(6.490)	(5.033)	(7.741)
**Large herbivore presence**	5.137	-3.459	-0.619	10.047 **	2.572	-3.288	5.724 *	-6.374	-1.084	0.971	1.003
	(4.376)	(3.742)	(4.588)	(4.165)	(5.925)	(3.571)	(3.135)	(7.810)	(4.944)	(3.321)	(4.815)
**-Cost (EUR)**	0.225	-0.222	-0.431 **	-0.722 ***	0.197	0.261	-0.146	-0.445	-0.014	0.063	0.014
	(0.272)	(0.209)	(0.195)	(0.211)	(0.315)	(0.240)	(0.175)	(0.286)	(0.275)	(0.206)	(0.226)

Table S3. Results of the MXL model: the case of Germany. Part 1: Voting behavior.

	**Random parameters**		**Voting: base level is CDU**						
	**Mean**	**Std. dev.**	**Wouldnt vote**	**SPD**	**GRUNE**	**AFD**	**FDP**	**LINKE**	**Other**
**ASC SQ**	-150.4 ***	155.7 ***	-5.889	-3.209	-34.2 ***	-0.836	-10.15	-75.65 ***	-42.55 ***
	(6.890)	(6.725)	(6.239)	(7.229)	(7.461)	(6.438)	(10.505)	(8.134)	(6.771)
**Forest (log)**	22.886 ***	40.372 ***	3.095	0.342	11.034 **	-3.542	-12.52 **	-10.1 **	-4.102
	(3.780)	(1.707)	(4.118)	(4.542)	(4.550)	(3.986)	(5.673)	(4.624)	(5.314)
**River (log)**	43.711 ***	53.758 ***	-13.05 ***	3.387	31.707 ***	0.625	-5.5	-3.777	1.209
	(3.138)	(1.890)	(3.351)	(4.739)	(3.802)	(3.364)	(4.839)	(3.978)	(4.735)
**Agriculture (log)**	37.03 ***	38.967 ***	-13.52 ***	-10.49 ***	12.143 ***	-2.826	-25.31 ***	-9.763 *	-2.096
	(3.405)	(1.688)	(3.853)	(3.826)	(3.583)	(4.180)	(6.086)	(5.384)	(4.540)
**Connectivity (log)**	53.576 ***	62.405 ***	-13.23 ***	9.087 **	19.111 ***	8.545 **	-16.35 ***	24.66 ***	0.702
	(3.392)	(2.534)	(3.539)	(4.462)	(5.161)	(3.796)	(6.255)	(4.616)	(4.401)
**Large carnivore presence**	52.869 ***	81.842 ***	-0.277	17.062 ***	8.623 ***	-7.43 **	-6.538	30.367 ***	4.427
	(3.285)	(2.530)	(3.177)	(3.698)	(3.106)	(3.188)	(5.845)	(4.168)	(3.730)
**Large herbivore presence**	41.605 ***	43.412 ***	-6.863 ***	-1.201	-3.87	-0.509	-1.737	-1.332	-0.113
	(2.484)	(1.850)	(2.068)	(2.447)	(2.533)	(2.225)	(3.066)	(2.831)	(3.261)
**-Cost (EUR)**	0.021 ***	1.905 ***	0.183	-0.027	0.111	-0.358 *	-0.239	0.801 ***	0.261
	(0.003)	(0.089)	(0.192)	(0.196)	(0.202)	(0.216)	(0.248)	(0.246)	(0.285)

Notes:

[1] ***, **, and * indicate 1%, 5%, and 10% significance levels, respectively.

[2] The model was estimated in WTP-space and then rescaled, so that the column "Mean" would represent the average WTP for the voter of CDU (base level) with all other characteristics at the mean level. The interaction effects with other political parties represent a deviation from the average WTP of CDU voter.

[3] Figure 2 in the main text is based on the results reported in this table.

[4] All coefficients, except for *Cost*, can be interpreted in monetary terms (EUR).

[5] *Cost*'s random parameter has a log-normal distribution. In column "Mean" we report a median of this distribution, whereas in column "Std. dev." we report the standard deviation of the underlying normal distribution. Interaction terms refer to the change in the mean of the underlying normal distribution as per formula (3).

Table S4. Results of the MXL model: the case of Germany. Part 2: Other effects.

								Regions: base level is Berlin		
	Distance (log)	Age	Male	City size	Income	Income missing	Education	Baden-Wurttemberg	Bayern	Brandenburg
**ASC SQ**	9.176	10.085	-1.412	4.573 **	-10.325 ***	-7.681	14.393 ***	13.506	17.596	24.39 **
	(19.424)	(14.647)	(4.044)	(1.818)	(1.527)	(9.029)	(5.215)	(31.443)	(27.307)	(10.103)
**Forest (log)**	-29.651 **	-12.95 *	-3.471	3.073 **	-1.772 *	-19.445 ***	-5.023 *	50.697 ***	60.931 ***	49.604 ***
	(12.429)	(7.635)	(2.237)	(1.228)	(0.908)	(5.982)	(3.014)	(19.385)	(16.741)	(5.888)
**River (log)**	20.856 **	34.386 ***	-1.949	1.114	1.447 *	-25.587 ***	4.669 *	-9.979	-15.282	16.736 ***
	(10.444)	(7.478)	(1.904)	(0.973)	(0.826)	(4.511)	(2.688)	(17.038)	(14.520)	(4.758)
**Agriculture (log)**	3.954	0.636	-3.2	2.365 **	1.156	-10.115 *	3.58	8.569	6.825	19.429 ***
	(10.981)	(7.612)	(2.128)	(1.085)	(0.867)	(5.699)	(2.995)	(17.708)	(14.763)	(6.211)
**Connectivity (log)**	0.732	57.5 ***	-8.844 ***	-1.373	-0.327	10.083 *	14.731 ***	17.912	9.019	7.75
	(11.298)	(8.073)	(2.230)	(1.090)	(0.951)	(5.555)	(3.160)	(17.919)	(15.525)	(5.263)
**Large carnivore presence**	10.197	18.728 ***	-6.497 ***	0.76	-0.61	22.038 ***	5.172 **	-4.222	0.939	3.092
	(9.397)	(6.981)	(1.737)	(1.040)	(0.909)	(4.088)	(2.550)	(15.094)	(12.714)	(4.458)
**Large herbivore presence**	-11.58 *	-21.385 ***	1.021	-2.274 ***	-0.927 *	8.237 **	2.233	5.072	9.611	-16.78 ***
	(6.836)	(4.780)	(1.307)	(0.652)	(0.523)	(3.360)	(1.776)	(10.890)	(9.399)	(3.354)
**-Cost (EUR)**	0.524	1.698 ***	0.026	0.116 **	-0.02	0.756	-0.417 ***	-0.932	-0.458	0.451
	(0.537)	(0.366)	(0.122)	(0.047)	(0.040)	(0.485)	(0.149)	(0.862)	(0.761)	(0.275)

Regions (continued): base level is Berlin									
	**Hamburg**	**Hessen**	**Mecklenburg-Vorpommern**	**Niedersachsen / Bremen**	**Nordrhein-Westfalen**	**Rheinland-Pfalz / Saarland**	**Sachsen / Thuringen**	**Sachsen-Anhalt**	**Schleswig-Holstein**
**ASC SQ**	-28.188	-0.16	18.659 *	13.931	10.694	14.151	2.995	-18.711	32.977 *
	(18.311)	(27.667)	(11.197)	(18.057)	(25.753)	(29.011)	(24.536)	(35.093)	(17.878)
**Forest (log)**	-10.331	57.911 ***	23.024 ***	37.814 ***	35.095 **	67.334 ***	42.282 ***	2.425	30.514 ***
	(11.973)	(17.087)	(8.717)	(10.692)	(16.250)	(18.134)	(15.139)	(13.395)	(10.047)
**River (log)**	8.931	-19.007	13.392 **	-4.043	-5.757	-16.603	-6.817	5.833	-1.158
	(9.561)	(13.391)	(6.234)	(10.260)	(13.200)	(16.092)	(11.123)	(18.700)	(9.680)
**Agriculture (log)**	23.732 **	-10.972	26.032 ***	8.753	3.831	1.714	25.9 *	-38.465 ***	-3.512
	(9.898)	(15.196)	(10.013)	(10.005)	(14.290)	(16.282)	(13.528)	(13.353)	(9.305)
**Connectivity (log)**	-14.618	-1.99	-10.769	7.095	-2.269	1.529	-2.55	3.503	22.842 **
	(10.325)	(16.687)	(6.931)	(10.581)	(14.647)	(17.332)	(13.904)	(20.993)	(10.397)
**Large carnivore presence**	-12.101	-16.038	2.747	-1.771	-7.811	-9.026	-7.587	2.176	12.827
	(8.082)	(12.908)	(5.626)	(9.504)	(12.832)	(14.542)	(10.562)	(17.057)	(8.822)
**Large herbivore presence**	-11.002	9.59	-6.587	9.91 *	20.328 **	9.456	-3.993	-3.067	5.885
	(8.049)	(9.220)	(6.030)	(5.973)	(9.387)	(10.317)	(7.442)	(8.658)	(6.063)
**-Cost (EUR)**	-0.5	-0.368	0.363	-0.084	-0.629	-0.416	-0.428	-0.387	0.158
	(0.531)	(0.743)	(0.309)	(0.549)	(0.690)	(0.821)	(0.577)	(0.570)	(0.435)

Notes:

[1] Several regions were merged based on the geographical proximity as there was not enough observations for some of them to achieve proper convergence (less than 1% of respondents). The names of the merged regions are separated with “/”.

Table S5. Summary of SQ choices and WTP values: the case of Poland.

	PIS		Wouldn't vote		KO		P2050		NL		PSL		KONF		Other	
**Share of SQ choices**	0.095	***	0.119	***	0.047	***	0.037	***	0.035	***	0.005	***	0.116	***	0.073	***
									**Willingness to pay**							
**Landscape (log)**	12.43	***	11.7	***	26.441	***	25.061	***	26.008	***	41.456	***	8.814	***	20.088	***
	(2.500)		(2.917)		(2.243)		(2.902)		(3.643)		(5.116)		(3.065)		(1.953)	
**Connectivity (log)**	50.639	***	54.697	***	59.521	***	54.108	***	70.04	***	22.048	***	45.753	***	46.864	***
	(4.166)		(6.127)		(4.129)		(5.288)		(6.451)		(6.906)		(5.097)		(3.515)	
**Animals**	64.353	***	57.689	***	68.123	***	52.265	***	68.753	***	59.058	***	68.486	***	59.314	***
	(2.993)		(3.800)		(2.847)		(2.969)		(3.729)		(4.467)		(3.617)		(2.480)	

Notes:

[1] ***, **, and * indicate 1%, 5%, and 10% significance levels, respectively.

[2] WTP for *Landscape* is the average of WTP for *Forest*, *River*, and *Agriculture*.

[3] WTP for *Animals* is the average of WTP for *Large carnivore presence* and *Large herbivore presence*.

[4] The values from this table are depicted in Figure 6.

Table S6. Summary of SQ choices and WTP values: the case of Germany.

	CDU		Wouldn't vote		SPD		GRUNE		AFD		FDP		LINKE		Other	
**Share of SQ choices**	0.211	***	0.255	***	0.149	***	0.082	***	0.242	***	0.15	***	0.092	***	0.171	***
							**Willingness to pay**									
**Landscape (log)**	34.542	***	26.719	***	32.289	***	52.837	***	32.628	***	20.099	***	26.664	***	32.879	***
	(2.477)		(2.457)		(2.524)		(2.459)		(2.358)		(3.261)		(2.855)		(2.991)	
**Connectivity (log)**	53.576	***	40.351	***	62.663	***	72.686	***	62.121	***	37.229	***	78.235	***	54.278	***
	(3.392)		(3.532)		(4.123)		(5.401)		(3.903)		(6.135)		(4.459)		(4.649)	
**Animals**	47.237	***	43.667	***	55.168	***	49.614	***	43.268	***	43.1	***	61.755	***	49.394	***
	(2.255)		(2.183)		(2.254)		(2.302)		(2.324)		(3.446)		(3.013)		(2.939)	

Notes:

[1] ***, **, and * indicate 1%, 5%, and 10% significance levels, respectively.

[2] WTP for *Landscape* is the average of WTP for *Forest*, *River*, and *Agriculture*.

[3] WTP for *Animals* is the average of WTP for *Large carnivore presence* and *Large herbivore presence*.

[4] The values from this table are depicted in Figure 7.
